# Supplementary material for: Single-cell multiomic profiling of human lungs reveals cell-type-specific and age-dynamic control of SARS-CoV2 host genes
Source: eLife. 2020 Nov 9;9:e62522. doi: 10.7554/eLife.62522 (PMC7688309; doi:10.7554/eLife.62522)
Supplement: Transparent reporting form [file elife-62522-transrepform.docx]

***eLife’s* transparent reporting form**

We encourage authors to provide detailed information *within their submission* to facilitate the interpretation and replication of experiments. Authors can upload supporting documentation to indicate the use of appropriate reporting guidelines for health-related research (see [EQUATOR Network](http://www.equator-network.org/%20)), life science research (see the [BioSharing Information Resource](https://biosharing.org/)), or the [ARRIVE guidelines](http://www.plosbiology.org/article/info:doi/10.1371/journal.pbio.1000412) for reporting work involving animal research. Where applicable, authors should refer to any relevant reporting standards documents in this form.

If you have any questions, please consult our Journal Policies and/or contact us: [editorial@elifesciences.org](mailto:editorial@elifesciences.org).

**Sample-size estimation**

- You should state whether an appropriate sample size was computed when the study was being designed
- You should state the statistical method of sample size computation and any required assumptions
- If no explicit power analysis was used, you should describe how you decided what sample (replicate) size (number) to use

Please outline where this information can be found within the submission (e.g., sections or figure legends), or explain why this information doesn’t apply to your submission:

No statistical methods were used to predetermine sample sizes. Information can be found in the method section “Statistics”.

**Replicates**

- You should report how often each experiment was performed
- You should include a definition of biological versus technical replication
- The data obtained should be provided and sufficient information should be provided to indicate the number of independent biological and/or technical replicates
- If you encountered any outliers, you should describe how these were handled
- Criteria for exclusion/inclusion of data should be clearly stated
- High-throughput sequence data should be uploaded before submission, with a private link for reviewers provided (these are available from both GEO and ArrayExpress)

Please outline where this information can be found within the submission (e.g., sections or figure legends), or explain why this information doesn’t apply to your submission:

For individual lung tissue samples one single nucleus ATAC-seq and RNA-seq library was generated. For one lung tissue (3 yo, donor D32), we performed a technical replicate for single nucleus ATAC-seq. Technical replicate refers to generation of another library from the same ground tissue sample.

Fore each time point experiments were performed for 3 individuals.

No sample was excluded from data analysis. For single nucleus ATAC-seq, we generated one additional dataset from a 4 month old lung which is part of the combined clustering.

Low quality nuclei and potential doublets were excluded from analysis as outlined in method sections “Single nucleus RNA-seq analysis” and “Single nucleus ATAC-seq analysis”.

Processed data are available via our web portal available at www.lungepigenome.org.

Raw sequencing files will be submitted to dbGAP.

**Statistical reporting**

- Statistical analysis methods should be described and justified
- Raw data should be presented in figures whenever informative to do so (typically when N per group is less than 10)
- For each experiment, you should identify the statistical tests used, exact values of N, definitions of center, methods of multiple test correction, and dispersion and precision measures (e.g., mean, median, SD, SEM, confidence intervals; and, for the major substantive results, a measure of effect size (e.g., Pearson's r, Cohen's d)
- Report exact p-values wherever possible alongside the summary statistics and 95% confidence intervals. These should be reported for all key questions and not only when the p-value is less than 0.05.

Please outline where this information can be found within the submission (e.g., sections or figure legends), or explain why this information doesn’t apply to your submission:

To compare fraction of positive cells between different age groups, a two-tailed unpaired t-test was used. For genome-wide differential accessibility analysis of snATAC-seq peaks, pairwise comparisons between donor age groups were made using EdgeR with a cutoff of FDR < 0.05. For locus restricted differential accessibility analysis of snATAC-seq peaks, pairwise comparisons between donor age groups (n=3 per age group) were made using independent t-test with same variance assumption. This information can be found in the method section “Statistics” and in related figure legends (Figure 3, Figure 3 – figure supplement 1).

To predict the effects of variants on chromatin accessibility in AT2 cells we applied deltaSVM algorithm. We calculated p-values and q-values and defined variants with significant effects using a threshold of FDR<0.1. This information can be found in the method section “Predicting variant effects on TF binding and chromatin accessibility” and in the related figure legend (Figure 4/5).

(For large datasets, or papers with a very large number of statistical tests, you may upload a single table file with tests, Ns, etc., with reference to sections in the manuscript.)

**Group allocation**

- Indicate how samples were allocated into experimental groups (in the case of clinical studies, please specify allocation to treatment method); if randomization was used, please also state if restricted randomization was applied
- Indicate if masking was used during group allocation, data collection and/or data analysis

Please outline where this information can be found within the submission (e.g., sections or figure legends), or explain why this information doesn’t apply to your submission:

Sample were allocated to groups based on age. There was no randomization and investigators were not blinded to the investigated specimens. This information can be found in the method section “Statistics”.

**Additional data files (“source data”)**

- We encourage you to upload relevant additional data files, such as numerical data that are represented as a graph in a figure, or as a summary table
- Where provided, these should be in the most useful format, and they can be uploaded as “Source data” files linked to a main figure or table
- Include model definition files including the full list of parameters used
- Include code used for data analysis (e.g., R, MatLab)
- Avoid stating that data files are “available upon request”

Please indicate the figures or tables for which source data files have been provided:

Count matrices for snRNA-seq and snATAC-seq and a full peak list are available for download on our web portal available at [www.lungepigenome.org](http://www.lungepigenome.org). We point to the portal at multiple parts of the manuscript (abstract, discussion and method section “Data availability”.

Source data for Figure 1 – figure supplement 1 is available as Supplementary Table 2; Source data for Figure 3B and Figure 3 – figure supplement 1A is available as Supplementary Table 3. Source data for Figure 3C is available as Figure 3–Source Data 1. Source data for Figure 3E is available as Supplementary Table 4. Source data for Figure 3F is available as Supplementary Table 5. Source data for Figure 3G is available as Supplementary Table 6.

Source data for Figure 4A is available as Supplementary Table 7.

The source data are referenced throughout the results section linked to the figure panel.

Custom code (other tools were used with default parameters or with modifications stated in the method section):

https://github.com/kjgaulton/pipelines/tree/master/lung_snATAC_pipelinehttps://gitlab.com/Grouumf/ATACdemultiplex/-/blob/master/scripts/DA_analysis_with_edgeR.R

https://gitlab.com/Grouumf/ATACdemultiplex/-/blob/master/scripts/snATAC_entropy_feature

https://gitlab.com/Grouumf/ATACdemultiplex/-/tree/master/ATACdemultiplex
